# Search for Spiritual Assistance in Religious Organizations: What Are the Motives of Persons Who Have Experienced Destructive Relationships at Work?

**DOI:** 10.3389/fpsyg.2021.702284

**Published:** 2021-09-21

**Authors:** Jolita Vveinhardt, Mykolas Deikus

**Affiliations:** ^1^Department of Management, Faculty of Economics and Management, Vytautas Magnus University, Kaunas, Lithuania; ^2^Department of Theology, Faculty of Catholic Theology, Vytautas Magnus University, Kaunas, Lithuania

**Keywords:** spiritual assistance, motives, religious organizations, victim, destructive relations between employees

## Abstract

Religious assistance is an effective tool in helping the victims of violence, but so far, it has not been investigated that what promotes the persons aggrieved in the workplace to seek such assistance. Therefore, this study aimed to identify the motives of persons who have experienced destructive relationships, which inspire the search for spiritual assistance in religious organizations, seeking to develop the provision of such service. After the analysis of the scientific literature, a questionnaire “Motives of Persons Aggrieved at Work for Seeking Spiritual Assistance (MP-SSA-40)” was developed and validated. In this study, a survey was conducted on 463 persons working in Lithuanian organizations using the questionnaire survey method. The correlation and regression analyses were performed. The study has shown that the religiosity of the victim is not the only condition for seeking spiritual assistance. Significant factors are the congruence of values borne by the person and the religious organization, the motives arising from the relation with the person providing assistance, and the motives determined by circumstances that create an important contextual background. The established trends show that by solving work-related problems and problems of interpersonal relationships of assistance providers, obstacles hindering the implementation of intrinsic motives of the victim to apply for spiritual assistance to the religious organization, providing it can be removed. The value of the article is demonstrated by the fact that it fills the existing gaps of knowledge about the motives of victims of destructive relationships at work, seeking religious spiritual assistance. In addition, a new and reliable instrument to measure such motives is proposed.

## Introduction

Analyzing the possibilities of assistance to the victims of the destructive behavior of coworkers in the scientific literature, in addition to the organizational factors (Mountjoy et al., 2016), and factors related to wider assistance of the social environment (Zapf, 1999; Duffy and Spery, 2014) are highlighted. On the other hand, victims, especially those who have experienced more severe forms of psychological and physical violence, need professional help (Hoel et al., 2002; Carnero, 2005; Carnero and Martinez, 2005; Duffy and Sperry, 2007; Ferris, 2009) in solving negative consequences for health, professional activity, and social well-being. In this context, in parallel with therapy, the emphasis is placed on the religious aspect (Pargament et al., 2011; Zenkert et al., 2014; Benson et al., 2016; Ruth-Sahd et al., 2018), as research shows that the application of religious resources and spiritual care measures not only helps to convalesce from a traumatic experience (Rambo, 2010) but also restores the integrity of broken relationships with the environment and ensures the intrinsic sense of well-being (Bartel, 2004; Delgado, 2005). Although it is observed that the commonality of religious values facilitates the relationship with the persons seeking assistance (Delgado, 2005); however, research on the possibilities of religious organizations to provide assistance to persons who have experienced destructive relationships at work is not abundant (Nunez and Gonzalez, 2009; Vensel, 2012; Turner, 2016, 2018). The lack of such research aggravates not only the possibilities to use potential resources of religious organizations in providing assistance to persons who have experienced psychological traumas in the workplace but also in explaining the motives of victims for which such assistance is sought or not sought. Therefore, the research problem is formulated by the question: What motives of persons who have experienced destructive relationships inspire the search for spiritual assistance in religious organizations and how to develop the provision of such service? The purpose of this study is to identify the motives of persons who have experienced destructive relationships, inspiring the search for spiritual assistance in religious organizations, and seeking to develop the provision of such service.

## Literature

### Destructive Interrelationships of Employees

Studies show that experienced violence, especially re-victimization, is one of the strongest sources of trauma (Mittal et al., 2021). In this context, destructive relationships at work, also called toxic, are a wide range of negative acts of violence manifesting themselves as bullying, intolerance, harassment in the workplace, and the like (Neuman, 2000; Goldman, 2008; Hollis, 2015; Milosevic et al., 2019). Research also emphasizes toxic employees (Jonason et al., 2012; Esaulova and Nagibina, 2017; Helfrich and Dietl, 2019) initiating destructive interrelationships, the most radical form of which is workplace mobbing—a phenomenon encompassing a wide range of consequences (Topa Cantisano et al., 2007; Kozakova et al., 2018; Pheko, 2018; Erdis et al., 2019). The victims experience strong emotional and/or physical suffering (Vartia, 2001; Duffy and Sperry, 2007; Merilainen et al., 2016), and in extreme cases—mobbing—they feel helpless and unable to defend themselves (Nielsen et al., 2015). The crises experienced at work negatively affect the career, family relationships, disturb the social lives of the victims, and require significant treatment costs (Carnero and Martinez, 2005; Duffy and Sperry, 2007). In addition, although consciousness, knowledge, and understanding of the people themselves are important in overcoming trauma resulting from workplace mobbing and bullying, many victims desire to be heard and need the therapeutic and support of friends (Pheko, 2018).

### Influence of Religiosity

A number of studies demonstrate that the religiosity of a person and compliance of values in relation to the institute providing assistance are significant criteria in making the decision to seek help. The study conducted in the United States has shown strong links among religious behavior, strength of faith, spirituality, and religious coping (Freiheit et al., 2006). In this case, only the search of believers for the assistance of God was investigated, and the study itself was conducted by surveying the students of the Catholic University (*N* = 124). In another case, however, Moreno and Cardemil (2013) have found that especially religious persons older in age preferred methods of religious and spiritual coping, primarily supporting the religious counseling services that would correspond to their religious beliefs (the United States qualitative study, *N* = 17). Besides, contextual influencing factors such as the already existing experience and more formal education were also identified. In principal, the trends repeated the results obtained in a larger sample (*N* = 235), which showed the link between a preference for religious assistance and the religiosity of younger individuals too in seeking to overcome the psychological suffering caused by the negative situations (Crosby and Bossley, 2012). At the same time, it was observed that higher religious spirituality was associated with the less frequent search for a mental health professional (*N* = 2,285) (Abe-Kim et al., 2004), although addressing the clergyman was not associated with dissatisfaction with the mental health system (Sørgaard et al., 1996). In any case, it is significant that both the faith and religious practices have a positive effect on self-esteem, interpersonal sensitivity, psychological wellbeing regardless of culture (Plante and Boccaccini, 1997; Pakpour et al., 2014; Cummings et al., 2015; Estrada et al., 2019), and the higher the religiosity and spirituality of a person, the greater the need to address not only a mental health professional but also individuals who specialize in the religious spiritual assistance field (Abe-Kim et al., 2004).

### Role of Values

In addition to religious beliefs, an important variable is culture-specific values and the relation with the religious organization. *On the one hand*, it is emphasized that religious values are related to the survival of the community and the assistance to its members. That is, persons attending religious community meetings can also expect more significant support from the community (Grayman-Simpson and Mattis, 2013a,b). However, not so much the very participation in the activities of communities but the relation becomes the decisive factor. Pessi (2013) distinguished the three main constituents of the model of the relation of individuals with the church: experience (rites, traditions, and emotions), values (both church expectations and personal expectations with regard to the church), and truth (presentation of religious activeness, frames of reflections, clear attitudes, and space for individuality). The connecting link is the authenticity of each of these three constituents. *On the other hand*, Rogers-Sirin et al. (2017) found that family values as an important factor influencing the negative relationship between religiosity and seeking psychological assistance (*N* = 496). The explanation is found in the incompatibility of traditional family values and values characteristic to psychotherapy. It is namely the cultural differences that promote doubts and discussion on whether the traditional models of psychotherapeutic assistance can be universally applied in different cultural and social group settings (Koç and Kafa, 2019). Therefore, given the importance of religious beliefs, both physicians are advised to consider the religious needs of patients (Dein, 2004; Saguil and Phelps, 2012) and representatives of religious communities are recommended to improve their spiritual care practices.

### Spiritual Assistance of Religious Organizations

Pastoral models of religious organizations place a significant emphasis on coping with the spiritual crisis caused by psychological trauma. Scientific literature uses several terms to describe the active actions and processes that include efforts to help persons experiencing various crisis situations after they have experienced physical and psychological traumas: *spiritual care* (Entwistle et al., 2018), *spiritual coping* (Hartwick and Kang, 2013); *spiritual counseling, pastoral care* (Hunsinger, 2011), and *spiritual accompaniment* (Gubi, 2011).

While in the process of spiritual guidance, it is avoided to advise the guided persons because there is a risk of diverting from what God wants to say to them (Hagmaier, 1963, p. 129), and pastoral care is indispensable to wise advice. As Hiltner (1949) observed, the purpose of pastoral advice can be seen as an attempt to help people understand their intrinsic conflicts. Another important aspect of pastoral care is that the pastor does not necessarily limit themselves to a conversation with the persons seeking help. According to Magezi (2019), pastoral care, or “Cura animarum” as the concept of “healing of the soul,” is related to care about the whole person. This expresses a holistic view to the person, viewing from the perspective of the incarnation of God and love of the loved one, responding to the needs of people, and the models of pastoral care can vary depending on time and context. Yet both the spiritual guidance and pastoral care are related to the same goal. Coping with spiritual pain, the focus is on restoring the interpersonal relationships, developing the relation with God in the Spirit, listening, sagacity, and spiritual growth (Burton, 2004, p. 3).

Solving the arising crises, the same methods of coping with the spiritual post-traumatic state are treated as the ones that have answered the purpose. These are the prayer of intercession and the personal prayer as regular communication with God (Deventer-Noordeloos and Sremac, 2018), involvement in the activities of the community (Schuster et al., 2001), reading of the Holy Scripture and meditation (Hartwick and Kang, 2013), and other methods. That is, the conception of spiritual coping is more a reflection of the “technique” or means used by individuals on their own or after receiving the advice of a pastor or counselor.

### Availability of Assistance

Although religious beliefs help to cope with the arisen psychological problems, far from all religious persons seek help from the person providing spiritual assistance. This is shown by the study of Larson and Larson (2003). Out of 80% of persons who used spiritual coping, only a little less than one-fifth sought direct help from a clergyman. A similar gap was found in the study conducted by Pinkard and Heflinger (2006), which also drew attention to the contribution of organizations themselves in attracting infrequent church attendees. Regardless of the possibilities of proposed assistance, other studies place an important emphasis on removing obstacles to information and appropriate guidance (Harris et al., 2001), on applied or generally insufficiently exploited marketing tools of pastoral care and counseling (Wrenn et al., 1995; Webb, 2012), which, according to Lageman (1984), should be oriented to the ones used in medicine. Nevertheless, research, although sparse, shows that religious organizations do not exploit their possessed potential in providing assistance to the victims of destructive relationships in the workplace (Hagmaier, 1963; Vensel, 2012; Turner, 2017).

After conducting a study in one of the United Kingdom dioceses, Turner (2018) distinguished the key obstacles making spiritual assistance unavailable: the lack of assistance, the wish of potential assistants to dissociate themselves from the victim, expecting benefit from concealing the event, etc. In addition, attention was drawn to the inconsistency of the organized assistance. Vensel (2012) pointed out that clergymen who had experienced mobbing and were trying to use an independent religious coping style often took the risk of experiencing the negative consequences of professional burnout. A study conducted in Lithuania showed that religious organizations did not use the existing possibilities to help victims aggrieved in the working environment due to the narrow specialization of their activity, from which the latter segment of clients “falls out,” and the lack of knowledge (Deikus, 2019).

### Factors Inspiring the Choice of Assistance

Research related to therapeutic assistance investigates intrinsic and extrinsic factors promoting decision-making (Zuroff et al., 2007; Ryan and Deci, 2008). According to Ryan and Deci (2000), the self-determination theory explains the causes of both passive and active behavior. This theory first draws attention to direct social contexts of individuals and then, to their developmental environment, as social and contextual conditions facilitate natural intrinsic motivation processes that are important in healthcare, religion, psychotherapy, etc. On the one hand, people want to be competent and autonomous and on the other hand, they want to be related. Social contexts in which people act, no matter whether they are close or distant (e.g., the cultural value), affect their type of motivation, influence well-being, and efficiency (Deci and Ryan, 2012).

The theory of intrinsic motivation, which states that people have innate motives, emphasizes the cognitive aspect too, as people choose what to do while handling information they receive from the environment and memory (Deci, 1975; Ryan and Deci, 2000). In addition to intrinsic and extrinsic motivation, Zuroff et al. (2007) emphasize integrated motivation that distinguishes itself by autonomy. This means that the person accepts the importance of the personal goal and integrates that goal into their core values and beliefs. In this context, Ren (2010) maintains that an important source of intrinsic motivation is value congruence that encourages the efforts of a person to act. According to the author, values affect the thinking of the people and it is believed that innate intrinsic motivation strengthens through value congruence. Frey (1994), meanwhile, emphasizes the importance of the relation in intrinsic motivation. The closer the relation between the two actors, the greater is the significance of a particular factor.

## Method

The conception of this study is based on the research into destructive relationships in the workplace and assistance to aggrieved persons by integrating the approach of religious spiritual assistance. No studies were found in scientific databases, analyzing the motives of victims of destructive relationships at work to seek help in religious organizations. For this reason, persons working in Lithuanian organizations were surveyed to find out the motives that encouraged them to seek religious spiritual assistance.

Prior to the start of the survey, it was estimated that at 95% probability and 5% error, the sample must include 384 respondents, because according to the data published by the Lithuanian Department of Statistics in 2018, the working-age population in Lithuania are 1,389,788 persons. The questionnaire was placed on the survey platform. A function of a mandatory answer was attributed to all the questions to avoid incorrect completion, i.e., questionnaires with unanswered questions or with incompletely answered questions. Therefore, the sample of this study did not contain questionnaires rejected due to incomplete or incorrect filling. Equal response ratings and completion of the questionnaire from the same IP address were also prohibited. Access to the questionnaire was not made public, and an electronic survey link was distributed to the randomly selected respondents in person by email.

The respondents were introduced to the purpose of the study, anonymity and confidentiality were guaranteed, and it was explained that the data collected would be used exclusively for scientific purposes. An informed consent was obtained from each participant. The survey was conducted in January–March, 2020 and involved 463 employed persons (from the age of 18 years to retirement age and employed persons of retirement age).

The formation of the research instrument involved several stages: (1) analysis of scientific articles published in the Web of Science Core Collection (Clarivate Analytics) (hereinafter, WoS), Scopus and EBSCO databases; (2) the analysis of research instruments adjacent to the analyzed topic; (3) preparation of the initial version of the research instrument (the instrument included four scales, eight subscales, and 64 items); (4) peer review of the research instrument (peer review involved four experts whose main selection criteria were as follows: a doctorate degree in social sciences and humanities, theology, psychology, marketing, and management fields obtained at least 5 years ago; scientific publications of the last 5 years corresponded, and/or were related to the topic under analysis); (5) revisions of the research instrument, considering the comments of experts (items with an average score of 3.5 or lower, where the maximum could have been 5 were rejected; items with the average score above 3.5 but not reaching 5 were revised); (6) the survey of the target population (10 persons [five men and five women] representing different age groups and professions, who have different educational backgrounds, were selected for the survey); (7) revisions of the research instrument, taking into account the observations of the participants of the targeted survey (revised wordings of items by changing the scientific language into the colloquial language that is understandable to the persons who have various educational backgrounds and professions); and (8) developed instrument for conducting the study “Motives of Persons Aggrieved at Work for Seeking Spiritual Assistance—MP-SSA-40”, the structure of the research instrument and its psychometric characteristics are presented in Table 1.

**Table 1:** Psychometric characteristics of the instrument “Motives of Persons Aggrieved at Work for Seeking Spiritual Assistance (MP-SSA-40)”: prime and secondary factorization.

Scales^*^	Subscales^**^	**Nofitems**	**Explained Dispersion, %**	**Cronbach alpha**	**Spearman-Brown**	**Factor Loading (L)**			**Total Item Correlation (r/itt)**			Principal components F1^***^	**Alpha factoring F1**
						**mean**	**min**.	**max**.	**mean**	**min**.	**max**.		
DR	DRA	7	54.92	0.86	0.82	0.74	0.65	0.79	0.54	0.33	0.80	0.89	0.75
	DRC	3	61.87	0.69	–	0.78	0.59	0.87	0.60	0.28	0.87	0.89	0.75
Explained Dispersion, %												78.48	56.86
VL	VLP	6	65.90	0.89	0.87	0.80	0.51	0.87	0.64	0.29	0.86	0.95	0.89
	VLO	6	64.64	0.89	0.83	0.80	0.72	0.86	0.64	0.40	0.86	0.95	0.89
Explained Dispersion, %												89.99	79.93
SA	SAK	6	67.66	0.90	0.86	0.82	0.74	0.89	0.67	0.46	0.88	–	–
MT	MTR	5	79.32	0.93	0.90	0.89	0.81	0.92	0.79	0.63	0.92	0.92	0.84
	MTC	7	61.50	0.90	0.89	0.78	0.74	0.85	0.61	0.44	0.84	0.92	0.84
Explained Dispersion, %												85.43	70.78

* *Scales: DR, Destructive relationships; VL, Values; SA, Service awareness; MT, Motives*.

** *Subscales: DRA, Destructive actions; DRC, Causes of destructive actions; VLP, Personal values; VLO, Values of religious organizations; SAK, Knowledge of assistance provided by religious organizations; MTR, Motives determined by the relation; MTC, Motives determined by circumstances*.

*** *Principal components (a single-factor model) F1*.

Destructive relationships include such categories of behavior manifesting itself in the working environment as harassment, mobbing, persecution (Rasool et al., 2020), unethical relationships (Atakan et al., 2008), bullying (Olafsson and Johannsdottir, 2004), and the like. According to Leymann (1990, 1996), a specific form of behavior, such as verbal and non-verbal ways of affecting the victim, stands out in this context—mobbing, which as an extreme stressor is distinguished according to the criteria of frequency (at least once a week) and duration of attacks (at least for half a year) (Leymann, 1990, 1996; Zapf and Kuhl, 2000). Therefore, in this study, it was intended to divide respondents into *three groups*: (1) who have experienced mobbing; (2) who have experienced destructive behavior but have not experienced mobbing; (3) who have not experienced either mobbing or destructive behavior.

## Results

For the purposes of this study, one of the criteria (I) was faith in a deity. The results showed that 79.5% of respondents were believers, the majority of whom were women-−86.4% (men-−69.8%). The distribution of respondents by age (five groups) and gender is presented in Table 2. The largest share of respondents that got into in the sample (72.8%) indicated that they belonged to the Roman Catholic community, which is in line with the trends seen in the Lithuanian census (i.e., in the census of 2011, 77.2% of the population attributed themselves to Roman Catholics; it should be noted that during the census, employed and non-employed persons were not distinguished). In addition, 4.1% belonged to the Evangelical Lutheran community; 1.6% attributed themselves to Pagans; 1.2% were Orthodox; 1% belonged to Christian Reformed Church communities; 0.6% belonged to each of Greek Rite Catholics and Buddhists; 0.4% were Judaeos; 0.2% to Anglicans. The rest did not belong to any community of faith or indicated to be agnostic. The results of the study show that the share of persons who have attributed themselves to non-believers or agnostics still have contacts with houses of worship in religious communities, which becomes clear due to visits to them. Despite the significant number of believers, less than one-tenth of the respondents participated in the liturgical rites, and just 5.6% actively participated in the activities of religious communities (Table 2).

**Table 2:**
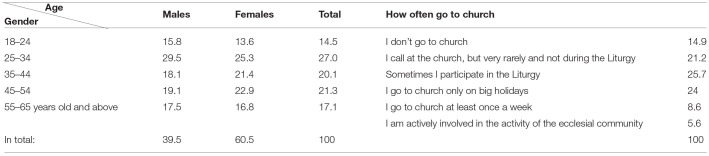
Distribution of respondents by age, gender and Church^*^ attendance, participation in Liturgy, and community activities, %.

* *To simplify the presentation of the results, the houses of worship of religious communities are referred to here as the “church”*.

Seeking the objectives of this study, one of the criteria (II) was experiences of destructive relationships at work. Approximately half (49.7%) of the respondents indicated that they had not experienced destructive behavior of the employees, while 6.8% of the survey participants noted behavior attributable to mobbing. The remaining part, that is, 33.5%t, experienced destructive relationships that are not attributed to mobbing, that is, when destructive type attacks recur less than once a week or for less than half a year.

Destructive relationships encourage the search for religious spiritual assistance, but a much more important factor here is religious values. Evans (1996) suggests for the absolute value of *r*: 0.00–0.19 (very weak correlation), 0.20–0.39 (weak correlation), 0.40–0.59 (moderate strength), 0.60–0.79 (strong correlation), and 0.80–1.0 (very strong correlation). Thus, a more detailed picture unfolds at the sub-scale level, where strong links are found among the personal, religious values, and motives determined by the relation, just as the approval of religious values is strongly linked with the motives related to the relation with the spiritual assistance provider (Table 3).

**Table 3:** Intercorrelation relationships between the subscales of experiences of destructive relationships at work and motives for seeking spiritual assistance in religious organizations (*N* = 463).

Subscales^***^	**DRA**	**DRC**	**VLP**	**VLO**	**SAK**	**MTC**	**MTR**
DRA	1	0.531^**^	0	0	0	0.186^**^	0.107^*^
		0.000	0.134	0.435	0.461	0.000	0.022
DRC	0.531^**^	1	0.149^**^	0.106^*^	0.152^**^	0.208^**^	0.163^**^
	0.000		0.001	0.023	0.001	0.000	0.000
VLP	0	0.149^**^	1	0.793^**^	0.579^**^	0.488^**^	0.709^**^
	0.134	0.001		0.000	0.000	0.000	0.000
VLO	0	0.106^*^	0.793^**^	1	0.620^**^	0.576^**^	0.757^**^
	0.435	0.023	0.000		0.000	0.000	0.000
SAK	0	0.152^**^	0.579^**^	0.620^**^	1	0.418^**^	0.622^**^
	0.461	0.001	0.000	0.000		0.000	0.000
MTC	0.186^**^	0.208^**^	0.488^**^	0.576^**^	0.418^**^	1	0.665^**^
	0.000	0.000	0.000	0.000	0.000		0.000
MTR	0.107^*^	0.163^**^	0.709^**^	0.757^**^	0.622^**^	0.665^**^	1
	0.022	0.000	0.000	0.000	0.000	0.000	

* *Correlation is significant at the 0.05 level (2-tailed)*.

** *Correlation is significant at the 0.01 level (2-tailed)*.

*** *Subscales: DRA, Destructive actions; DRC, Causes of destructive actions; VLP, Personal values; VLO, Values of religious organizations; SAK, Knowledge of assistance provided by religious organizations; MTC, Motives determined by circumstances; MTR, Motives determined by the relation*.

*Values of intercorrelation relationships: 0.6 < r ≤ 0.8 strong, 0.4 < r ≤ 0.6 moderate, 0.2 < r ≤ 0.4 weak, 0.1 ≤ r ≤ 0.2 very weak*.

However, correlations show only the strength of relationships; therefore, to determine the relationship of the variables, seven linear regression equations were constructed, based on the results of calculations presented in Table 4.

**Table 4:** Relationships between the subscales of experiences of destructive relationships at work and of motives for seeking spiritual assistance in religious organizations (*N* =4 63).

	**Dependent variable**															
	DRA				DRC				VLP				VLO			
	*R*	*R^2^*	*R^2^_*r*_*	*P*	*R*	*R^2^*	*R^2^_*r*_*	*p*	*R*	*R^2^*	*R^2^_*r*_*	*p*	*R*	*R^2^*	*R^2^_*r*_*	*p*
	0.587	0.344	0.336	0	0.592	0.35	0.341	0	0.821	0.675	0.67	0	0.857	0.735	0.732	0
IV	nB*	sB^**^	t	A p^***^	nB^*^	sB^**^	t	A p^***^	nB^*^	sB^**^	t	A p^***^	nB^*^	sB^**^	t	A p^***^
*Constant*	0.951		7.262	0	0.38		3.531	0	0.328		2.755	0.006	0.46		4.551	0
DRA					0.515	0.554	14.282	0	0.008	0.006	0.191	0.849	−0.038	−0.032	−1.085	0.278
DRC	0.6	0.558	14.282	0					0.052	0.039	1.178	0.24	−0.037	−0.029	−0.973	0.331
VLP	0.01	0.013	0.191	0.849	0.059	0.078	1.178	0.24					0.464	0.486	13.651	0
VLO	−0.068	−0.08	−1.085	0.278	−0.056	−0.071	−0.973	0.331	0.626	0.597	13.651	0				
SAK	−0.083	−0.096	−1.827	0.068	0.118	0.147	2.822	0.005	0.083	0.079	2.118	0.035	0.153	0.151	4.595	0
MTC	0.133	0.164	2.94	0.003	0.046	0.061	1.085	0.278	−0.071	−0.07	−1.784	0.075	0.159	0.167	4.782	0
MTR	0.007	0.009	0.129	0.898	−0.031	−0.045	−0.645	0.519	0.232	0.255	5.376	0	0.171	0.198	4.578	0
	SAK					MTC						MTR				
	*R*		*R^2^*	*R^2^_*r*_*	*p*	*R*		*R^2^*	*R^2^_*r*_*		*p*	*R*		*R^2^*	*R^2^_*r*_*	*p*
	0.698		0.487	0.48	0	0.738		0.545	0.539		0	0.838		0.703	0.699	0
IV	nB^*^		sB^**^	t	A p^***^	nB^*^		sB^**^	t		A p^***^	nB^*^		sB^**^	t	A p^***^
*Constant*	0.614			4.404	0	0.918			6.788		0	0.684			5.589	0
DRA	−0.088		−0.075	−1.827	0.068	0.14		0.114	2.94		0.003	0.006		0.004	0.129	0.898
DRC	0.146		0.116	2.822	0.005	0.056		0.042	1.085		0.278	−0.03		−0.02	−0.645	0.519
VLP	0.117		0.124	2.118	0.035	−0.098		−0.098	−1.784		0.075	0.257		0.233	5.376	0
VLO	0.289		0.293	4.595	0	0.3		0.286	4.782		0	0.256		0.222	4.578	0
SAK						0		0	−0.002		0.998	0.219		0.187	5.422	0
MTC	0		0	−0.002	0.998							0.394		0.357	10.516	0
MTR	0.277		0.323	5.422	0	0.495		0.547	10.516		0					

*R^2^_r_,revised; p, reliability*.

* *nB, unstandardized Beta coefficient;*

** *sB, standardized Beta coefficient;*

*** *A p, ANOVA reliability. IV, Independent variable*.

*Subscales: DRA, Destructive actions; DRC, Causes of destructive actions; VLP, Personal values; VLO, Values of religious organizations; SAK, Knowledge of assistance provided by religious organizations; MTC, Motives determined by circumstances; MTR, Motives determined by the relation*.

DRA = 0.587 + 0.600 ^*^ DRC + 0.133 ^*^ MTC.

DRC = 0.380 + 0.515 ^*^ DRA + 0.118 ^*^ SAK.

VLP = 0.328 + 0.626 ^*^ VLO + 0.083 ^*^ SAK + 0.232 ^*^ MTR.

VLO = 0.460 + 0.464 ^*^ VLP + 0.153 ^*^ SAK + 0.159 ^*^ MTC + 0.171 ^*^ MTR.

SAK = 0.614 + 0.146 ^*^ DRC + 0.117 ^*^ VLP + 0.289 ^*^ VLO + 0.277 ^*^ MTR.

MTC = 0.918 + 0.140 ^*^ DRA + 0.300 ^*^ VLO + 0.495 ^*^ MTR.

MTR = 0.684 + 0.257 ^*^ VLP + 0.256 ^*^ VLO + 0.219 ^*^ SAK + 0.394 ^*^ MTC.

The results of the regression analysis show several trends. *First*, when each of the causes of destructive relationships at work and motives determined by circumstances are individually increasing and the remaining variables remain unchanged, the variable of destructive relationships at work is also increasing. In other words, when negative actions are experienced more often, the reasons will become more apparent and circumstances will become more important, even if awareness of the assistance and personal values and approval of values of religious organizations remain the same. *Second*, as the actions of destructive relationships at work and awareness of assistance provided by religious organizations are increasing separately one-by-one, while the rest of the variables remain unchanged, the variable of causes of destructive relationships at work is also increasing. *Third*, when variables, such as values of religious organizations, awareness of assistance provided by religious organizations, and motives determined by the relation are increasing individually, one-by-one and the remaining variables are not changing, the variable of motives determined by circumstances is also increasing. That is, the circumstances determining the search for religious spiritual assistance will become more important along with the increasing approval of values of religious organizations and awareness of assistance. Of course, this does not mean that personal values or experiences are not important, but this emphasizes the role of the religious organization and objectives raised to it. *Fourth*, as the variables of personal values, the assistance provided by religious organizations, motives determined by circumstances, and motives determined by the relation are increasing individually one-by-one and the rest are not changing, the variable of values of religious organizations is also increasing. This demonstrates that even if the flow of information about the provided spiritual assistance is not increased and the situation of destructive relationships remains the same, approval of the values of religious organizations may increase. *Fifth*, along with the increase of variables of causes of destructive relationships at work, personal values, values of religious organizations, and motives determined by the relation individually one-by-one, while the rest of the variables remain unchanged, the variable of awareness of assistance provided by religious organizations is also increasing. That is to say, along with the increase of the above-mentioned variables, attention to information about the possibilities of provided assistance may be increasing. *Sixth*, when the variables of actions of destructive relationships at work, values of religious organizations, and motives determined by the relation are increasing individually one-by-one, while the remaining variables are not changing, the variable of motives determined by circumstances is increasing. This shows that in this case, both the causes of the negative relationships and awareness of the assistance are not that significant. *Seventh*, as the variables of personal values, values of religious organizations, the assistance provided by religious organizations, and motives determined by circumstances are individually increasing one-by-one, while the remaining variables are not changing, the value of the independent variable of motives determined by the relation is increasing. Again, the latter variable is not significantly influenced by service awareness, experiences of negative relationships, and their causes.

## Discussion and Conclusion

Studies conducted in other countries show that spiritual assistance, religious coping are a sufficiently efficient way of coping with consequences of various crises (Schuster et al., 2001; Sauter, 2004; Pargament et al., 2011; Moreno and Cardemil, 2013), and the number of employees believing in God (79.5%), identified in Lithuania, even with the error of 5% gives a reason to hope that the development of religious spiritual assistance can be quite a significant support for persons aggrieved in the workplace. However, several significant circumstances have been identified, which may aggravate the possibilities to receive such assistance. The unfolded gap between faith in God and rare visits to the house of prayer, engagement in the Liturgy, and other activities of the religious community and low emphasis on religious teaching suggest that religiosity is more associated with the individual relation with the deity than with the community of believers as such. The identified trends of the relation with the religious community basically confirm the results of other studies conducted in Lithuania. For example, the majority of respondents in the study conducted by Navaitis et al. (2014) were persons who visit houses of prayer on the big holidays, and those who regularly go to church and participate in the community activities constituted the least share (slightly more than 5%). Despite the unfolded individualized relationship with the deity, a particularly strong and statistically reliable relationship between personal values and values of the religious organization was established. Value approaches and their congruence are significant in several respects. *First*, the similarity of values ensures a closer connection with the person providing assistance, making it easier to establish a contact (Saguil and Phelps, 2012). It follows that, *second*, the value-based relationship strengthens the attractiveness of religious spiritual assistance. This suggests that in the context of the intrinsic motivation theory (Zuroff et al., 2007; Ren, 2010), value congruence plays a particularly important role in making a decision to seek assistance in the religious organization. However, the potential for community assistance cannot be exploited until the person feels expected in the community and can expect assistance (about two-thirds of respondents do not feel this), although the level of community assistance is identified in research as highly significant and efficient (Hunsinger, 2011; Abbott, 2012; Entwistle et al., 2018). This way, the indifferent behavior of the religious community can be treated as a specific hindering context (Ryan and Deci, 2000, 2008; Deci and Ryan, 2012).

The similarity of values, shared positive experience, and the guarantee of confidentiality are the triad of the main motives encouraging the search for spiritual assistance. Research shows that the assurance of safety in the context of spiritual assistance is an important value (Erde et al., 2006; Puchalski et al., 2009) that is demonstrated by this study too. Furthermore, it can be stated that low trust in the religious organization and unsuccessful personal relation with the clergyman or the clergy remains a strong obstacle for the development of spiritual assistance. In other words, if this problem is not solved, even a more abundant flow of information about the possibilities of spiritual assistance will hardly have a greater impact on the decisions to seek help from the religious organization. All the more so as particularly weak (albeit statistically significant) intercorrelational relationships were identified between the knowledge of provided spiritual assistance and the motives determined by circumstances. Meanwhile, the relationship between awareness and motives determined by the personal relation, on the contrary, was moderately strong, while the connection between the relation with the person providing assistance and the circumstances was extremely strong and statistically reliable. In addition, the results of the study demonstrate that negative experience is not that factor that would motivate a more intense search for information about provided religious spiritual assistance or would be more related to identification at the value level.

This study highlights the incentives of persons who have experienced destructive relationships in the workplace to seek religious spiritual assistance and shows a certain hierarchy of motivating factors. The scientific literature usually focuses on the religiosity of a person and the similarity of values borne by the person seeking assistance and by the one providing it; however, the obtained new knowledge enables to better understand what subjective and objective circumstances have a greater or lesser impact. Although the religiosity of the person and the similarity of values between the person and the religious organization are important factors, they are not the only ones. Significant influence is made by the relation with the person providing spiritual assistance and circumstances forming the contextual background, which may disturb the search for spiritual assistance. Although there are trends of individualized relation with God, which descend to religious practice as well, a greater obstacle to the possibility of developing spiritual assistance is the experiences of personal relationships with the religious organization and its representatives. The fact that the person does not feel being expected in the religious community or unmet expectations about the experienced interpersonal relation with the clergyman can become a strong argument for refusing to seek spiritual assistance. In this case, the situation is little affected by the nature of the coworkers' behavior that the person has experienced, while the identified certain lack of information about the provided assistance moves to the background. This allows religious organizations to reconsider their strategies of providing spiritual assistance at two levels: *first*, when making decisions related to the context hindering the search for help, and *second*, to direct attention for improving competence and interpersonal relationships of the persons providing spiritual assistance.

## Limitations and Future Research

There are several limitations to this study. Of course, a better understanding of the motives of persons seeking religious spiritual assistance requires further empirical research. Especially, considering that this study did not investigate how religious organizations of specific denominations operate (e.g., parishes, public institutions, communities, and other organizations). For example, Turner (2018) highlighted the shortcomings of availability of spiritual assistance to the clergy who experienced mobbing at the diocesan level, but it would not be less meaningful to investigate the situation at the level of a particular organization in terms of provision of assistance to the laity. This study reflects only the extent of information about the spiritual assistance provided by the religious organization and does not show the extent to which the assistance itself is provided. In addition, the commitment of the respondents themselves to religious organizations was not distinguished. Such analysis could be included in further research. As the percentages of trust in the religious organization and the experiences of the previous relationship with the clergyman or the clergy essentially coincided, it would make sense to analyze these relationships more thoroughly in the future. As homogeneous groups of respondents did not emerge, the analysis by religions was not performed. Nevertheless, we believe that the research findings still remain relevant as they provide a generalized picture of motives that are generally significant to the victims themselves. In the future, the analysis of assistance provided by religious organizations would enable to compare the situation by different religious denominations. In addition, it could be possible to investigate the impact of the attitude of victims toward the origin of their experienced illness; for example, misunderstanding of the will of God, the intervention of demonic forces (Pargament et al., 2011). Besides, the attitude of persons providing spiritual assistance toward the origin of illness would be also important (Leavey, 2010). Such data would be useful for both the religious organizations providing assistance and the psychotherapists, psychiatrists working outside them. The nature of religious assistance was also not detailed in this study, as we have not found any data on Christian certified counselors, Christian psychotherapists or Christian psychiatrists working in religious organizations. However, in the future, it would make sense to investigate the attractiveness of the assistance of such professionals to the victims who have suffered from destructive interpersonal relationships in the workplace. Although generally the low level of trust in the Church is associated with both scandals and secularism approaches, the question arises to what extent these tendencies can be offset or strengthened by the quality of the personal relationship with the clergy and the religious organization. This is a process related to two-way relationships, as the quality of the relationship and the assistance provided can also influence the growth of trust in the religious organization; however, the assessment of the strength of these relationships requires additional research.

## Data Availability Statement

The raw data supporting the conclusions of this article will be made available by the authors, without undue reservation.

## Ethics Statement

The studies involving human participants were reviewed and approved by Research Committee of the Faculty of Economics and Management of Vytautas Magnus University (Approval number: EVF-M-2020-01). The ethics committee waived the requirement of written informed consent for participation.

## Author Contributions

Both authors participated and contributed to study design, data collection, analysis and interpretation, writing, and original draft preparation.

## Funding

This research was funded by the European Social Fund under the No 09.3.3-LMT-K-712-16-0144 ‘Development of Competences of Scientists, other Researchers and Students through Practical Research Activities' measure.

## Conflict of Interest

The authors declare that the research was conducted in the absence of any commercial or financial relationships that could be construed as a potential conflict of interest.

## Publisher's Note

All claims expressed in this article are solely those of the authors and do not necessarily represent those of their affiliated organizations, or those of the publisher, the editors and the reviewers. Any product that may be evaluated in this article, or claim that may be made by its manufacturer, is not guaranteed or endorsed by the publisher.
