# Supplementary material for: Search for Spiritual Assistance in Religious Organizations: What Are the Motives of Persons Who Have Experienced Destructive Relationships at Work?
Source: Front Psychol. 2021 Sep 21;12:702284. doi: 10.3389/fpsyg.2021.702284 (PMC8490614; doi:10.3389/fpsyg.2021.702284)
Supplement: Supplementary file 1 [file Data_Sheet_1.pdf]

frontiers

Questionnaire

Motives of Persons Aggrieved at Work for Seeking Spiritual Assistance (MP-SSA-40)

Using the questionnaire in your research, please provide the reference to the following source:

Vveinhardt J and Deikus M (2021) Search for Spiritual Assistance in Religious Organizations: What Are the Motives of Persons Who Have Experienced Destructive Relationships at Work? Front. Psychol. 12:702284. doi: 10.3389/fpsyg.2021.702284

Scales, Subscales and Items	
DR. Destructive relationships	***DRA. Destructive actions***
	DRA1. I felt lonely because others held aloof from me, avoided communicating
	DRA2. I was wrongly accused, which worsened my reputation in the eyes of others
	DRA3. I was deliberately assigned tasks, being aware that I will not be able to accomplish them
	DRA4. Work was hampered (information was concealed, items were damaged, and the like)
	DRA5. I experienced gossips, rumours were spread about me
	DRA6. They yelled at me, used offensive words
	DRA7. I was threatened with violence and/or it was used
	DRC. Causes of destructive actions
	DRC1. I was bullied because of my appearance, health state, language, gait
	DRC2. There was mockery at my moral values, beliefs
	DRC3. I experienced hints and/or actions of a sexual nature
VL. Values	***VLP. Personal values***
	VLP1. Reading the Holy Scripture is important in my life
	VLP2. God is important in my life
	VLP3. It is important for me to get involved in the activities that the house of worship invites me to
	VLP4. A prayer, meditation, religious reflections are important in my life
	VLP5. It is important to me to follow everything the house of worship teaches in life
	VLP6. It is important for me to do good deeds without expecting a reward for this
	VLO. Values of religious organisations
	VLO1. I get all the answers to arising questions about the meaning of life
	VLO2. The house of worship provides every kind of assistance to those who need it
	VLO3. In my religious community, I feel I belong to it and I am expected

Scales, Subscales and Items	
	VLO4. I trust everything the house of worship preaches and does
	VLO5. I approve of what is said about moral problems and human needs
	VLO6. I approve of what is being taught about relationships based on love of the loved one
SA. Service awareness	***SAK. Knowledge of assistance provided by religious organisations***
	SAK1. It fell to me to read (hear) about it in the religious media
	SAK2. This was discussed at the gatherings of the religious community
	SAK3. The clergyman has spoken about it at worship and other events
	SAK4. It fell to me to read ads at a religious community home
	SAK5. It fell to me to read (hear) about it in the secular media
	SAK6. I heard familiar people speaking about it
MT. Motives	***MTC. Motives determined by circumstances***
	MTC1. I would address if those to whom helped personally recommended
	MTC2. I would address because confidentiality would be guaranteed (it will not be disclosed to anyone)
	MTC3. I would address if I didn’t know where to look for help
	MTC4. I would address if other professionals didn’t help.
	MTC5. I would address if the person providing assistance were attractive by his values
	MTC6. I would address because the service is free
	MTC7. I would address if I knew that the persons providing assistance have special education (psychological, spiritual counselling, spiritual accompaniment, etc.)
	MTR. Motives determined by the relation
	MTR1. I would address because previous experience in the relationships with the house of worship encourages trust in it
	MTR2. I would address because I believe the house of worship is merciful and helps everyone
	MTR3. I would address because I think it would help me to solve existential issues, issues of meaning of life
	MTR4. I would rely on the public recommendation of the clergyman
	MTR5. Stories read in the press about those who received similar assistance would help me to make a decision
